# Adjusting key performance indicators in ART laboratories according to controlled ovarian stimulation protocol

**DOI:** 10.3389/fphys.2025.1632623

**Published:** 2025-08-12

**Authors:** Wei Chen, Shiyuan Cheng, Yan Liu, Zhaoting Wu, Fuli Zhang, Xianju Huang, Guidong Yao, Wenyan Song, Haixia Jin

**Affiliations:** ^1^ Center for Reproductive Medicine, The First Affiliated Hospital of Zhengzhou University, Zhengzhou, China; ^2^ Henan Key Laboratory of Reproduction and Genetics, The First Affiliated Hospital of Zhengzhou University, Zhengzhou, China

**Keywords:** embryo laboratory, quality control, key performance indicators (KPIs), controlled ovarian stimulation (COS) protocol, blastocyst development

## Abstract

**Background:**

Reliable key performance indicators (KPIs) have been proposed to facilitate the quality management of embryo laboratories. However, the effects of controlled ovarian stimulation (COS) protocols on KPIs have not been adequately explored. This study aims to assess the variation in KPIs across four different COS protocols.

**Methods:**

We retrospectively analyzed 51,728 IVF/ICSI cycles in our center between 2014 and 2022. They were divided into four groups based on COS protocols: follicular phase long-acting (FPL) GnRH-a long, luteal phase short-acting (LPS) GnRH-a long, GnRH-ant, and micro-stimulation protocol groups. Several KPIs were compared across the four groups, and the results were validated in three age bands (<35 years, 35–38 years, and >38 years). Multivariable regression analysis was conducted to assess the effect of COS protocols on KPIs.

**Results:**

1) Significant differences were observed among the four groups in total blastocyst development rate (BLR) (56.2%, 54.1%, 49.7%, 29.5%, p < 0.001), total good blastocyst development rate (GBLR) (26.8%, 30.4%, 21.1%, 13.1%, p < 0.001), and implantation rate (46.6%, 30.7%, 24.2%, 13.1%, p < 0.001). 2) The LPS GnRH-a group showed a higher total GBLR (30.4%, 26.8%, p < 0.001) but a lower implantation rate compared to the FPL GnRH-a group (30.7%, 46.6%, p < 0.001). 3) Significant differences in KPIs were observed across all age groups, with the largest differences seen in women under 35 years 4) Multivariate logistic regression analysis revealed that these KPIs were significantly associated with the COS protocol.

**Conclusion:**

COS protocols significantly impact KPIs, suggesting that explore reliable KPIs according to treatment protocols could better evaluate the laboratory efficacy and provide a theoretical basis for personalized strategies for patients.

## 1 Introduction

Quality management in assisted reproductive technology (ART) laboratories is directly linked to embryo development and pregnancy outcomes. The introduction of data quality control management is not only essential for improving ART but also critical for advancing the field as a whole (Mayer et al., 2003). For instance, a previous study by Scaravelli and Giulia demonstrated that fertilization rate could serve as a key performance indicator (KPI) associated with the cumulative live birth rate (Scaravelli et al., 2021), both monitoring extrinsic factors affecting IVF embryo development and predicting clinical outcomes.

The establishment of KPIs for ART laboratories, which can evaluate the efficiency of embryologists and define the ideal reference range for each KPI, has long been discussed. The Association of Clinical Embryologists (ACE) created an international consensus that provided a set of IVF laboratory KPIs with suggested benchmarks (Hughes and Association of Clinical Embryologists, 2012). Subsequently, the Vienna Consensus (Embryology and Medicine, 2017) proposed specific competence levels and benchmark values for twelve KPIs, five performance indicators, and two reference indicators. Unlike the ACE guidelines, the Vienna Consensus also included Day 2 or 3 embryo development rate, successful biopsy rate, and implantation rate as KPIs while reclassifying the IVF/ICSI 1PN rate and IVF polyspermy rate as PIs.

Recently, the SIFES-MR and SIERR panels of experts delineated an updated set of KPIs that span both clinical and laboratory dimensions, such as the rate of cycles with moderate-to-severe ovarian hyperstimulation syndrome (OHSS) (Vaiarelli et al., 2023). Additionally, they developed a Center Performance Score formula to facilitate a comprehensive evaluation (Vaiarelli et al., 2023). Furthermore, researchers have been investigating novel KPIs, with prior studies suggesting that the Day 5 Usable Blastocyst Rate (D5BUR) (Hammond and Morbeck, 2019) and the number of MII oocytes required to produce a clinically usable embryo (Chamayou et al., 2021) can serve as innovative KPIs for quality control in the embryology laboratory.

To enhance the monitoring of clinical laboratory performance, it is essential to stratify the reference population for specific KPIs. For example, the Italian Consensus (Vaiarelli et al., 2023) has stratified the reference population based on female age, ovarian response, and the use of preimplantation genetic testing for aneuploidies (PGT-A). Carlotta (Zacà et al., 2022) et al. have further argued that KPIs related to extended embryo culture should be adjusted for advanced maternal age. These studies have been instrumental in improving quality control in embryo laboratories by enabling early detection of anomalies during *in vitro* culture, allowing for timely intervention and preventing issues from escalating.

Although the KPIs proposed in previous consensus documents are reliable, robust, and convenient to collect, experts have indicated that these KPIs could be further extended and refined (Hughes and Association of Clinical Embryologists, 2012; Embryology and Medicine, 2017). Threshold values for indicators included in a Total Quality Improvement plan should be based on their specific impact on outcomes and the protocols used in the laboratory (Mayer et al., 2003). Furthermore, previous research has emphasized that each center should select indicators based on laboratory organization and processes (Hughes and Association of Clinical Embryologists, 2012; Embryology and Medicine, 2017; De los Santos et al., 2016).

Controlled ovarian stimulation (COS) is a critical component in vitro fertilization-embryo transfer (IVF-ET), aiming to synchronize the development of multiple follicles. Over the past few decades, various COS protocols have been introduced, differentiating in terms of the patient population, medication timing, and associated costs. The main protocols include the luteal phase short-acting (LPS) gonadotropin-releasing hormone agonist (GnRH-a) long protocol, the follicular phase long-acting (FPL) GnRH-a long protocol, the GnRH antagonist (GnRH-ant) protocol, the short GnRH-a protocol, the micro-stimulation protocol, and the progestin-primed ovarian stimulation (PPOS) protocol. No single “optimum” protocol exists that meets all patients’ needs, and individualized protocols are highly recommended. Numerous studies have compared pregnancy outcomes and embryo quality across different protocols, but their effects on ART laboratory KPIs have not been adequately explored.

This study aims to explore reliable KPIs that reflect variation across different COS protocols, thereby facilitating the evaluation of laboratory performance. To date, no established KPIs have been tailored to COS protocols. There is limited evidence on this topic. This paper will compare the KPIs across four COS protocol groups and critically evaluate their validity and feasibility. The findings may contribute to further research on treatment protocols, provide a theoretical basis for personalized COS protocols for different patients, and offer novel suggestions for forecasting ART laboratory outcomes.

## 2 Methods

### 2.1 Patients and study design

**FIGURE 1:**
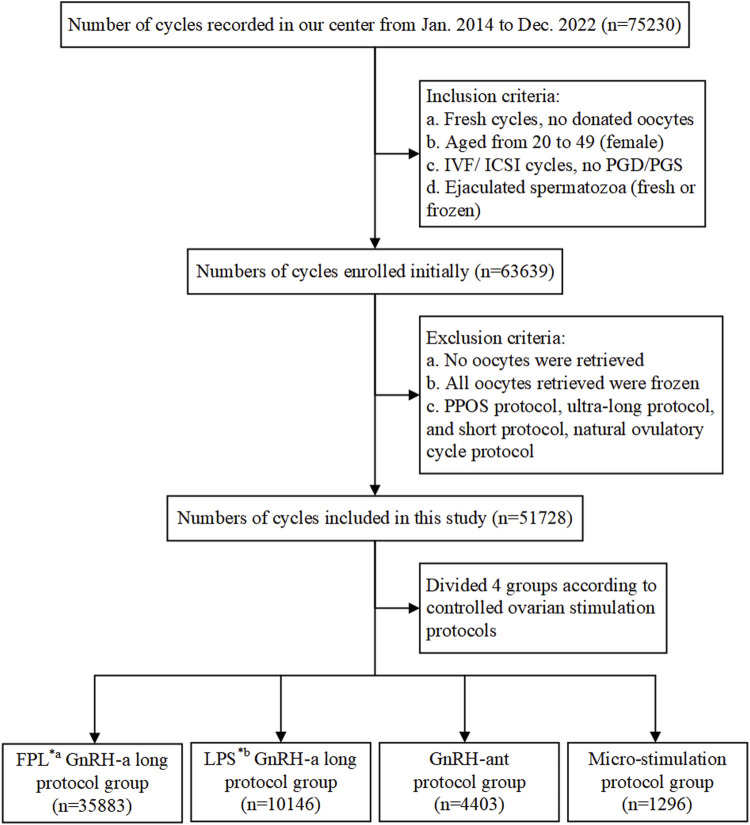
Flowchart showing data selection process and cycle distribution. Notes: IVF, *in vitro* fertilization; ICSI, intracytoplasmic sperm injection; PGD/PGS, preimplantation genetic diagnosis/screening; GnRH-a/ant, gonadotropin-releasing hormone agonist/antagonist; *a FPL, follicular phase long-acting; *b LPS, luteal phase short-acting.

We retrospectively analyzed medical data prospectively collected from infertile patients who underwent IVF treatment between January 2014 and December 2022 at the Reproductive Medicine Center of the First Affiliated Hospital of Zhengzhou University, Henan, China. As shown in the flowchart (Figure 1), 63,639 cycles initially met the inclusion criteria, and a total of 51,728 cycles were included in our study.

The inclusion criteria were as follows: (a) fresh IVF cycles; (b) female patients aged 20–49 years; (c) routine IVF or ICSI cycles, excluding preimplantation genetic diagnosis (PGD) or preimplantation genetic screening (PGS) cycles; (d) use of ejaculated spermatozoa, whether fresh or frozen. Cycles involving donor sperm or surgically retrieved sperm were excluded from the study. Additionally, cycles were excluded if: (a) no oocytes were retrieved; (b) all retrieved oocytes were frozen for specific reasons; (c) treatment protocols with a small number of cases, including the PPOS protocol, ultra-long protocol, short protocol, or natural ovulatory cycle protocol, were used.

Patients included in the study were categorized into four groups based on the treatment protocols they received: the FPL GnRH-a long protocol group (hereinafter referred to as FPL GnRH-a group, n = 35,883), the LPS GnRH-a long protocol group (hereinafter referred to as LPS GnRH-a group, n = 10,146), the GnRH-ant protocol group (n = 4,403), and the micro-stimulation protocol group (n = 1,296). The flowchart in Figure 1 details both the inclusion and exclusion criteria, as well as the distribution of cycles.

All patients were informed of the potential use of their medical records, and the study was approved by the Ethics Committee of the First Affiliated Hospital of Zhengzhou University.

### 2.2 COS protocols and IVF procedures

Controlled ovarian stimulation (COS) is a critical component of IVF-ET technology, aiming to produce an optimal number of follicles developing synchronously while minimizing the risk of OHSS through the careful administration of exogenous gonadotropins at specific points in the menstrual cycle. In our center, the decision regarding which COS protocol to use is based on several factors, including patient age, body mass index (BMI), causes of infertility, ovarian reserve function, and previous response to ovulation induction drugs.

**FIGURE 2:**
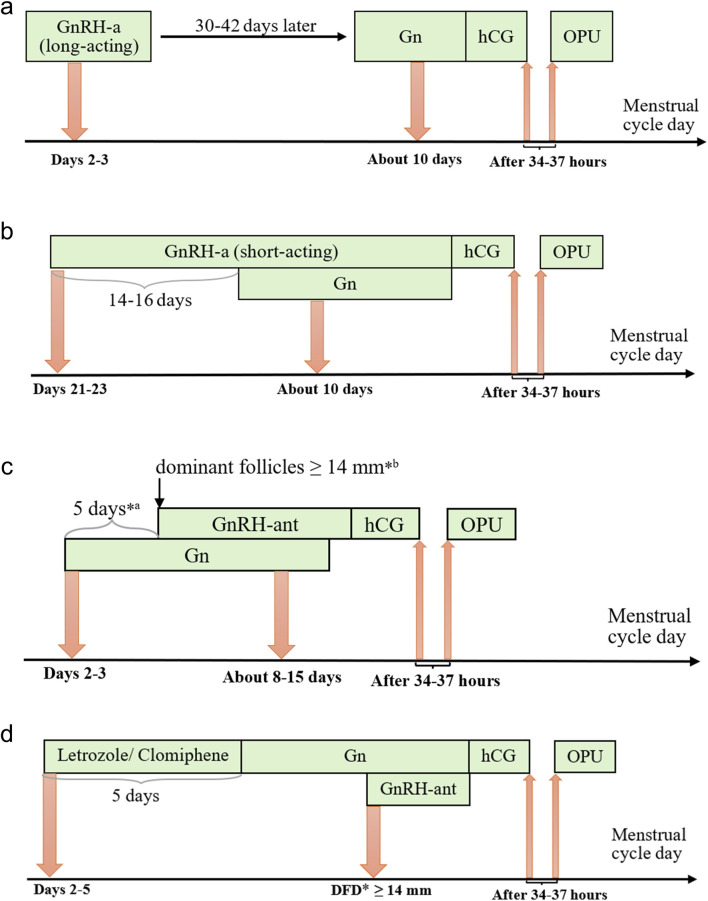
Protocols of controlled ovarian stimulation. Notes: **(a)** Long-acting GnRH-a protocol of the follicular phase, **(b)** Short-acting GnRH-a protocol of the luteal phase, **(c)** GnRH-ant protocol, and **(d)** Micro-stimulation protocol. Gn, gonadotropins; GnRH-a/ant, gonadotropin-releasing hormone agonist/antagonist; hCG, human chorionic gonadotropin; OPU, ovum pick-up; *a fixed protocol; *b flexible protocol; *DFD, dominant follicle diameter.

The treatment protocols adopted for the patients included in this study were the FPL GnRH-a long protocol, the LPS GnRH-a long protocol, the GnRH-ant protocol, and the micro-stimulation protocol. These four protocols differ in terms of drug selection, dosage, and timing of administration, and the standard procedures for each will be outlined below, briefly shown in Figure 2.

In the FPL GnRH-a long protocol, which has become increasingly prevalent in our center, a long-acting GnRH agonist (3.75 mg, Daphne, Ipsen, France) is administered on Day 2 or 3 of the menstrual cycle. After 30–42 days of downregulation, ovulation induction with gonadotropins (Gonal-f, Merck) is initiated when the following criteria are met: no follicles >10 mm in diameter, estradiol levels <183 pmol/L, and luteinizing hormone (LH) < 3 IU/L. The initial gonadotropin dose is determined based on the patient’s age, BMI, anti-Müllerian hormone (AMH) level, and previous ovarian response to COS and is adjusted during the course of stimulation according to hormone levels and follicular size. Human chorionic gonadotropin (hCG) (2000 IU, Livzon, China) and recombinant hCG (250 μg, Ovidrel, Merck, Italy) are administered when the following criteria are met: one dominant follicle ≥20 mm in diameter, at least three follicles ≥17 mm, or two-thirds of follicles ≥16 mm.

In the LPS GnRH-a long protocol cycle, a short-acting GnRH agonist (0.1 mg, Decapetyl, Ferring GmbH, Germany) is administered to patients during the mid-luteal phase (Days 21–23) of the preceding menstrual cycle. After 14–16 days, daily injections of gonadotropins (Gonal-f, Merck) are initiated when the patients meet the criteria for down-regulation, confirmed by ultrasound and serum hormone levels, as follows: endometrial thickness ≤5 mm; all follicles ≤5 mm in diameter; estradiol <183 pmol/L; FSH <5 IU/L; LH < 5 IU/L. Human chorionic gonadotropin (hCG) (2000 IU, Livzon, China) and recombinant hCG (250 μg, Merck, Italy) are administered when the follicle diameter reaches 18–20 mm.

In the GnRH-ant protocol cycle, recombinant FSH (112.5–300 IU, Puregon, Organon, The Netherlands) is administered to patients on Day 2 or 3 of the menstrual cycle. A daily injection of GnRH-ant (0.25 mg, Cetrotide, Pierre Fabre, France) is then performed using either a fixed or flexible protocol. In the fixed protocol, GnRH-ant is administered 5–6 days after ovulation triggering with gonadotropins, while in the flexible protocol, it is administered when the dominant follicles reach 14 mm in diameter. Adjustments to the gonadotropin dose, as well as the addition of FSH and exogenous LH, are made based on the estrogen levels in the follicles. Human chorionic gonadotropin (hCG) (2000 IU, Livzon, China) and recombinant hCG (250 μg, Merck, Italy) are injected when the following criteria are met: one dominant follicle ≥20 mm in diameter, two follicles ≥18 mm in diameter, or three follicles ≥17 mm in diameter.

In the micro-stimulation protocol cycle, Letrozole (5 mg/day, HENGRUI, China) or Clomiphene (100 mg/day, Codal, Cyprus) is administered orally to patients from Days 2–5 of the menstrual cycle for 5 days, followed by the administration of gonadotropins (Urofollitropin, Merck). Based on practical experience, GnRH-ant is added during the subsequent COS when the dominant follicle reaches a diameter of ≥14 mm. Monitoring is performed using vaginal ultrasound in combination with serum hormone levels, and human chorionic gonadotropin (hCG) (2000 IU, Livzon, China) and recombinant hCG (250 μg, Merck, Italy) are administered when the follicle diameter reaches 16–18 mm.

Ovum pick-up (OPU), guided by transvaginal ultrasound, was performed 34–37 h after hCG injection, with the day of OPU designated as Day 0. The oocyte-corona-cumulus complexes (OCCCs) identified under a stereoscopic microscope were immediately transferred to G-MOPS Plus (Vitrolife, Sweden) or K-SIGB (Cook, Australia) medium, washed three times in G-IVF Plus (Vitrolife, Sweden) or K-SIFM (Cook, Australia), and then placed in G-IVF Plus or K-SIFM medium in an incubator at 37°C with 6% CO2 and 5% O2 until insemination. Conventional IVF or ICSI was selected based on semen quality before and after oocyte denudation.

In a standard IVF cycle, semen was optimized using density gradient centrifugation and direct swim-up techniques, carried out 39–40 h after hCG injection. The optimized semen was then added to 50 µL drops of G-IVF Plus or K-SIFM medium, with 10,000 sperm added per oocyte. After co-culturing with optimized sperm for 5 h, the OCCCs were denuded using a denudation pipette (150 µm). Following denudation, the oocytes were cultured in G-IVF Plus or K-SIFM medium until the second polar body extrusion was observed, at which point the zygotes were transferred to G-1 Plus (Vitrolife, Sweden) or K-SICM (Cook, Australia) medium.

In an ICSI cycle, matured oocytes were denuded prior to insemination. The injected oocytes were placed in a cleavage culture medium, and fertilization was assessed 16–18 h after insemination. Normal fertilized oocytes were cultured in a cleavage medium until Day 3. On Day 3, embryos were evaluated based on morphology and were either cryopreserved, transferred, discarded, or extendedly cultured in G2 Plus (Vitrolife, Sweden) or K-SIBM (Cook, Australia) medium at 37°C with 6% CO2 and 5% O2 until Day 5/6.

Cleavage embryos and blastocysts were evaluated according to the Peter scoring system (Davis and Brinsden, 1999) and the Gardner scoring criteria (Gardner et al., 2000). Embryos fertilized *in vitro* were handled differently based on their morphology observed on Day 3 (Shen et al., 2024). Retained embryos were graded and prioritized for transfer, contributing to laboratory quality control. All patients received the same luteal support from the day of oocyte retrieval to 65 days post-embryo transfer.

### 2.3 Outcome measures

In this study, embryos scoring I or II according to the Peter scoring system were considered top-quality embryos, and blastocysts with a Gardner score ≥ 3BB were classified as high-quality blastocysts. Key performance indicators (KPIs) related to fertilization included the normal fertilization rate for IVF and ICSI. These rates were calculated by dividing the number of oocytes with 2PN (two pronuclei) and 2 PB (two polar bodies) by the number of COCs (cumulus-oocyte complexes) inseminated for IVF or MII (metaphase II) oocytes injected for ICSI, respectively.

Embryo quality indicators included the embryo development rate (top Day 3), good embryo development rate (top Day 3), GBLR (top Day 5/total), and total GBLR (top Day 5/total). The top Day 3 good embryo rate was calculated by dividing the number of top-quality embryos on Day 3 by the number of 2PN zygotes. The GBLR was determined by dividing the number of blastocysts by the number of embryos cultured to the blastocyst stage. The high-quality blastocyst rate was calculated by dividing the number of high-quality blastocysts by the number of embryos cultured in the blastocyst stage.

The primary clinical outcome measure was the implantation rate, which was assessed for both cleavage-stage embryos and blastocysts. The implantation rate was calculated as the number of gestational sacs detected via transvaginal ultrasound divided by the number of embryos transferred.

### 2.4 Statistical analysis

The COS groups mentioned above were compared in terms of female age, male age, maternal BMI, number of oocytes retrieved, duration of infertility, basal hormone levels, and the KPIs primarily discussed in this study. Continuous variables were expressed as mean ± SD and compared using either one-way ANOVA or the Kruskal-Wallis non-parametric test, depending on the data distribution. Comparisons of KPIs between the four COS groups and among age subgroups were performed using the chi-square test. Differences were considered statistically significant at p < 0.05 and highly significant at p < 0.01. Data were assessed after splitting cycles into four COS groups.

**FIGURE 6:**
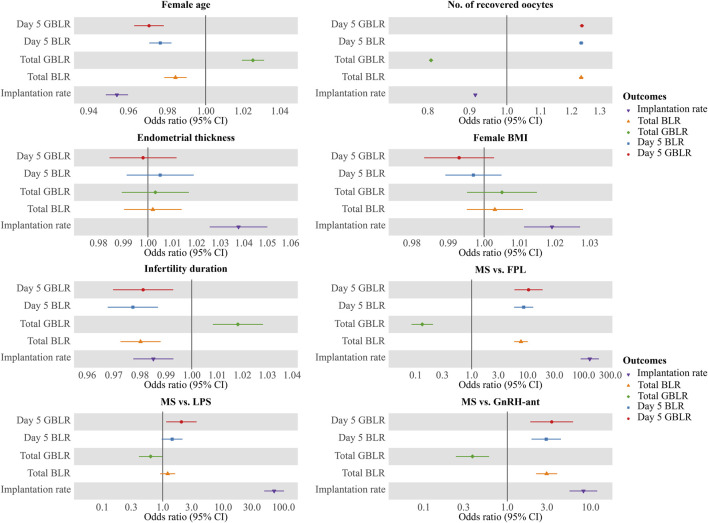
Multivariate logistic regression analysis of factors potentially impacting KPIs. Notes: Results of multiple logistic regressions of KPIs against female age, number of recovered oocytes, duration of infertility, endometrial thickness, and COS protocols (MS vs FPL, MS vs LPS, and MS vs GnRH-ant). KPIs here include Day 5 BLR, Day 5 GBLR, total BLR, total GBLR, and implantation rate. FPL, follicular phase long-acting GnRH-a protocol; LPS, luteal phase short-acting GnRH-a protocol; GnRH-ant, GnRH-ant protocol; MS, micro-stimulation protocol; BLR, blastocyte development rate; GBLR, good blastocyte development rate; 95% CI, 95% confidence intervals.

To evaluate the impact of the COS protocol on KPIs, stratified analyses were performed by sub-analyzing KPIs across three age bands. Multivariable regression analysis (MLR) was conducted to assess the effect of COS protocols and cycle characteristics on the KPIs. Covariates included female age, number of recovered oocytes, endometrial thickness, female BMI, infertility duration, and COS protocol (which included four categories). All data were obtained from the electronic medical record system of our reproductive center. Statistical analyses were performed using IBM SPSS Statistics 25. The results of MLR were represented in a forest plot (Figure 6) using the R package “ggforestplot”. Statistical graphs were drawn by GraphPad Prism 8.

## 3 Results

### 3.1 Patient characteristics

This study included 51,728 complete IVF/ICSI cycles performed at our center to comprehensively explore the effects of treatment protocols on embryo laboratory KPIs. The baseline characteristics of the four study groups are summarized in Table 1. Female age, the number of recovered oocytes, and male age were similar in the FPL and LPS GnRH-a groups, as well as in the GnRH-ant protocol and micro-stimulation groups, indicating no significant differences within these paired groups. However, there were significant differences between the four groups overall.

**TABLE 1:** Cycle characteristics of study groups.

Characteristic	Total n = 51,728	FPL^a^ GnRH-a protocol group n = 35,883	LPS^b^ GnRH-a protocol group n = 10,146	GnRH-ant protocol group n = 4,403	Micro-stimulation protocol group n = 1,296	*p-*value
Female age (year)	32.22 ± 5.64	31.29 ± 6.30	32.52 ± 4.79	37.18 ± 5.73	38.96 ± 5.50	<0.001
No. Of recovered oocytes	12.22 ± 7.65	13.75 ± 7.22	11.09 ± 7.03	5.31 ± 6.85	2.03 ± 2.08	<0.001
Male age (year)	33.09 ± 6.09	32.16 ± 5.45	33.48 ± 6.42	37.90 ± 7.07	39.53 ± 6.35	<0.001
Female BMI (kg/m^2^)	23.11 ± 2.90	23.11 ± 3.28	22.93 ± 3.14	23.60 ± 3.13	23.09 ± 2.90	<0.001
Infertility duration (year)	4.25 ± 3.15	4.05 ± 2.88	4.51 ± 3.23	4.91 ± 3.99	5.59 ± 4.58	<0.001
Endometrial thickness (mm)	12.04 ± 2.15	12.22 ± 2.24	11.62 ± 2.07	11.69 ± 1.56	11.78 ± 1.05	<0.001
Basal FSH (mIU/mL)	7.32 ± 3.56	6.75 ± 2.37	7.45 ± 3.04	9.98 ± 5.85	13.26 ± 9.66	<0.001
Basal estradiol (pg/mL)	8.40 ± 3.56	8.38 ± 2.51	8.48 ± 3.97	8.38 ± 6.50	8.18 ± 8.05	<0.001
Basal progesterone (ng/mL)	0.61 ± 1.37	0.55 ± 1.20	0.81 ± 1.87	0.55 ± 1.23	0.63 ± 1.53	<0.001
Basal LH (mIU/mL)	5.80 ± 4.73	5.85 ± 4.72	5.50 ± 4.24	5.81 ± 5.38	6.59 ± 5.90	<0.001
AMH (ng/mL)	3.33 ± 2.57	3.69 ± 2.68	2.91 ± 1.67	1.75 ± 2.70	2.04 ± 1.50	<0.001
Duration of Gn (days)	12.73 ± 2.74	13.61 ± 2.26	11.62 ± 2.07	9.76 ± 2.52	7.33 ± 3.65	<0.001
Dosage of Gn (IU)	2,597.78 ± 1,081.36	2,667.07 ± 1,086.85	2,478.23 ± 1,042.10	2,726.61 ± 854.67	1,177.50 ± 816.49	<0.001
LH on hCG day (mIU/mL)	1.76 ± 2.87	0.95 ± 1.13	1.85 ± 1.05	5.43 ± 4.95	10.79 ± 7.41	<0.001
Estradiol on hCG day (pg/mL)	3,271.05 ± 2,288.93	3,255.84 ± 1958.45	4,361.68 ± 2,813.56	1,690.44 ± 2,109.33	524.01 ± 697.32	<0.001
progesterone on hCG day (ng/mL)	0.98 ± 0.71	1.05 ± 0.65	0.91 ± 0.57	0.69 ± 0.70	0.84 ± 1.94	<0.001

Note.

^a^ FPL, follicular phase long-acting.

^b^ LPS, luteal phase short-acting.

The basal FSH level was significantly higher in the micro-stimulation group (13.2 ± 9.66 mIU/mL) compared to the other groups (p < 0.001). Other baseline characteristics, including duration of infertility, endometrial thickness, basal estradiol, basal progesterone, basal LH, and serum AMH levels, were similar across groups, although statistically significant differences were observed.

LH levels on the hCG day were significantly lower in the FPL and LPS GnRH-a groups (0.95 ± 1.13 mIU/mL and 1.85 ± 1.05 mIU/mL, respectively) compared to the other two groups (5.43 ± 4.95 mIU/mL and 10.79 ± 7.41 mIU/mL, respectively). Conversely, estradiol levels on the hCG day were higher in the FPL and LPS GnRH-a groups (3,255.84 ± 1958.45 pg/mL and 4,361.68 ± 2,813.56 pg/mL, respectively), followed by the GnRH-ant protocol group (1,690.44 ± 2,109.33 pg/mL) and the micro-stimulation protocol group (524.01 ± 697.32 pg/mL).

The duration of Gn administration, Gn dosage, and progesterone levels on the hCG day differed significantly across the four groups (p < 0.001 for all comparisons).

### 3.2 Outcomes of KPIs

Table 2 Outcomes of KPIs differed significantly between the COS protocol groups (Table 2). A complete account of these results is provided below.

**TABLE 2:** ART Comparison of KPIs between the four study groups.

Key performance indicators	Total n = 51,728	FPL*^1^ GnRH-a protocol group n = 35,883	LPS*^2^ GnRH-a protocol group n = 10,146	GnRH-ant protocol group n = 4,403	Micro-stimulation protocol group n = 1,296	*p-*value
ICSI normal fertilization rate	72.7%	74.1%^b^	70.3%^a^	70.7%^a^	66.8%^a^	<0.001
IVF normal fertilization rate	79.5%	79.5%^a^	79.9%^a^	79.2%^a^	74.2%^b^	<0.001
Day3 good embryo development rate	66.7%	66.9%^a^	66.8%^a^	64.3%^b^	60.3%^c^	<0.001
Day5 blastocyst development rate	32.6%	32.8%^a^	32.6%^a^	28.0%^b^	15.6%^c^	<0.001
Day5 good blastocyst development rate	21.3%	21.4%^a^	22.0%^a^	17.2%^b^	8.7%^c^	<0.001
Total blastocyst development rate	55.7%	56.2%^b^	54.1%^a^	49.7%^c^	29.5%^d^	<0.001
Total good blastocyst development rate	26.9%	26.8%^b^	30.4%^a^	21.1%^c^	13.1%^d^	<0.001
Implantation rate	42.0%	46.6%^b^	30.7%^a^	24.2%^c^	13.1%^d^	<0.001
Implantation rate (cleavage-stage)	39.3%	43.5%^b^	30.3%^a^	22.9%^c^	13.3%^d^	<0.001
Implantation rate (blastocyst-stage)	63.5%	65.5%^b^	43.6%^a^	43.1%^a^	0.0%^a,b^	<0.001

Note: Groups that share the same letter superscripts are not statistically different and groups denoted with different letters indicate statistically significant difference. *^1^ FPL, follicular phase long-acting; *2 LPS, luteal phase short-acting.

#### 3.2.1 Fertilization KPIs

Fertilization rates for routine *in vitro* fertilization (IVF) and intracytoplasmic sperm injection (ICSI) were evaluated across the four study groups. The ICSI fertilization rate in the FPL GnRH-a group (74.1%) was significantly higher than in the other three groups (p < 0.001). However, ICSI fertilization rates were comparable between the other three groups.

In contrast, IVF fertilization rates did not show significant differences among the FPL GnRH-a group, LPS GnRH-a group, and GnRH-ant protocol group (79.5%, 79.9%, and 79.2%, respectively). The micro-stimulation protocol group had a significantly lower IVF fertilization rate (74.2%, p < 0.001).

#### 3.2.2 Cleavage and blastocyst KPIs

Good embryo development rates on Day 3 were comparable in the FPL GnRH-a group (66.9%) and LPS GnRH-a group (66.8%), with statistically significant differences observed when compared to the GnRH-ant protocol group (64.3%) and micro-stimulation protocol group (60.3%) (p < 0.001).

Total GBLR differed significantly across all four study groups (56.2%, 54.1%, 49.7%, and 29.5%, p < 0.001), with the most notable decrease in the micro-stimulation protocol group. Similarly, the total GBLR showed statistically significant differences between the arbitrary pairs of groups (26.8%, 30.4%, 21.1%, and 13.1%, p < 0.001).

Regarding Day 5 BLR and GBLR, no significant differences were observed between the FPL GnRH-a group and the LPS GnRH-a group. However, significant differences were found between the GnRH-ant protocol group and the micro-stimulation protocol group. Specifically, statistical differences were evident between the arbitrary pairs of groups studied, except for the FPL and LPS GnRH-a groups.

#### 3.2.3 Implantation KPIs

Implantation rates were also evaluated for cleavage-stage and blastocyst-stage embryos across the four COS groups. The overall implantation rate was highest in the FPL GnRH-a group (46.6%) and lowest in the micro-stimulation protocol group (13.1%). The rates in the other two groups were 30.7% and 24.2%, respectively. Significant differences were observed between arbitrary two of our study groups (p < 0.001). Cleavage-stage implantation rates followed similar trends (Table 2).

Interestingly, while implantation rates for the blastocyst stage remained highest in the FPL GnRH-a long protocol group (65.5%), the rates were comparable between the LPS GnRH-a and GnRH-ant protocol groups (43.6% and 43.1%, respectively). In the micro-stimulation protocol group, only two patients aged 33 opted for blastocyst-stage embryo transfers, both of whom failed to conceive, resulting in a zero-implantation rate.

### 3.3 KPIs and female age

**FIGURE 3:**
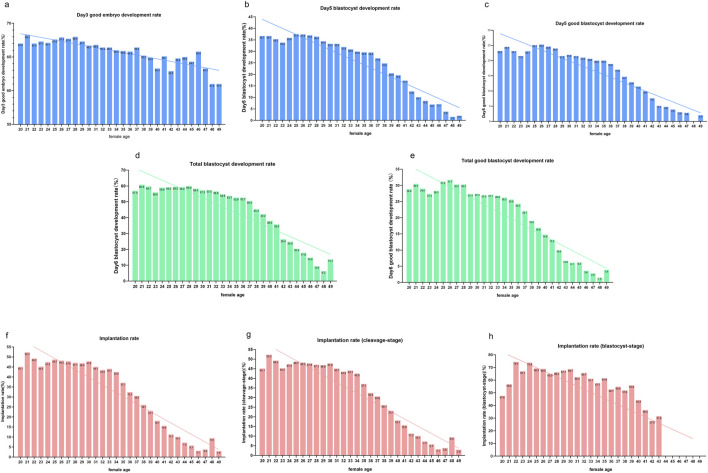
KPIs in cycles of different female ages. Notes: Profile of KPIs’ changes across four COS protocols in women aged from 20 to 49 years. KPIs here include **(a)** Day 3 good embryo development rate, **(b)** Day 5 blastocyte development rate (BLR), **(c)** Day 5 good blastocyte development rate (GBLR), **(d)** total BLR, **(e)** total GBLR, **(f)** implantation rate, **(g)** implantation rate (cleavage-stage), and **(h)** implantation rate (blastocyst-stage).

The results from the preliminary analysis of KPIs in relation to female age are presented in Figure 3. Although the patterns of decline varied slightly across the KPIs, all six KPIs showed a decline with advancing age. Overall, Day 3 good embryo development rates decreased steadily, with instability observed after age 38. Implantation rates and KPIs related to embryo and blastocyst development also declined with increasing age, with a pronounced drop observed at age 38. In contrast to cleavage-stage implantation rates, blastocyst-stage implantation rates remained more stable, with relatively high implantation rates maintained even beyond age 38.

### 3.4 KPIs and COS protocol

**FIGURE 4:**
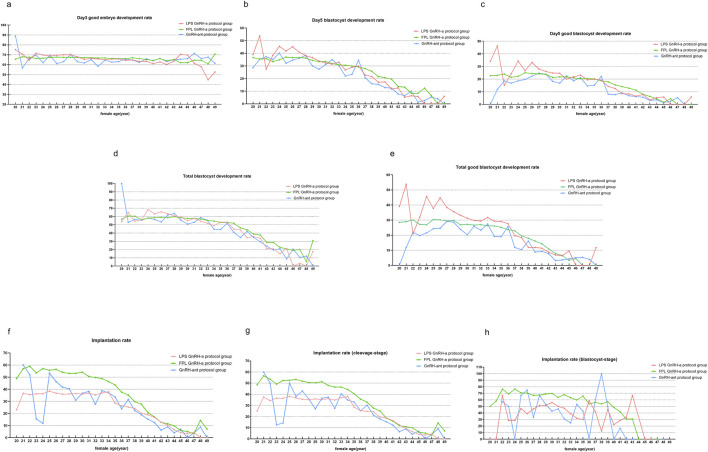
KPIs compared under different COS protocols. Notes: Comparisons of KPIs across three COS protocols in women aged from 20 to 49 years. KPIs here include **(a)** Day 3 good embryo development rate, **(b)** Day 5 blastocyte development rate (BLR), **(c)** Day 5 good blastocyte development rate (GBLR), **(d)** total BLR, **(e)** total GBLR, **(f)** implantation rate, **(g)** implantation rate (cleavage-stage), and **(h)** implantation rate (blastocyst-stage). GnRH-a/ant, gonadotropin-releasing hormone agonist/antagonist; FPL, follicular phase long-acting; LPS, luteal phase short-acting.

Figure 4 illustrates the intercorrelations among the three treatment protocols across different age groups. Since the study included a reduced number of cases using the micro-stimulation protocol at several age points, the changes in indicators by age are not provided. Day 3 good embryo development rates remain similar in FPL GnRH-a group, LPS GnRH-a group and GnRH-ant group across most age points. And the Day 5 BLR/GBLR and total BLR/GBLR were higher in the LPS GnRH-a group than in the FPL GnRH-a group for patients under 36 years old. Interestingly, the opposite trend was observed in cycles of patients older than 36 years, where these KPIs related to the blastocyst development were higher in the FPL GnRH-a group compared to the LPS GnRH-a group. Implantation rates for cycles in the FPL GnRH-a group were the highest across all age groups, with the most pronounced difference observed in women under 35 years old.

### 3.5 KPIs sub-analyzed according to COS protocol and female age

**FIGURE 5:**
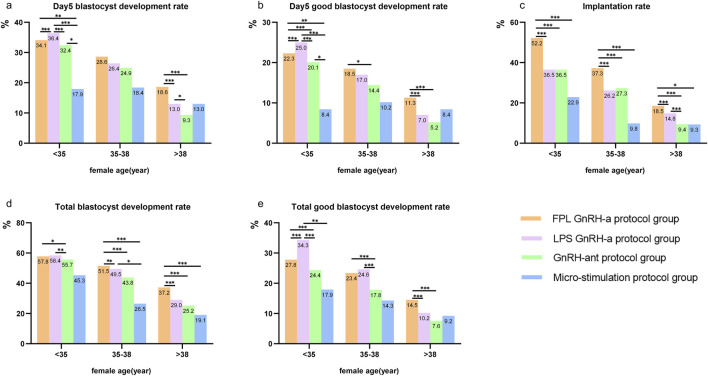
KPIs sub-analyzed according to COS protocol and female age. Notes: Comparisons of KPIs across four COS protocols in three age bands (<35 years, 35–38 years, and >38 years). KPIs here include **(a)** Day 5 blastocyte development rate, **(b)** Day 5 good blastocyte development rate, **(c)** implantation rate, **(d)** total blastocyte development rate, and **(e)** total good blastocyte development rate. GnRH-a/ant, gonadotropin-releasing hormone agonist/antagonist; FPL, follicular phase long-acting; LPS, luteal phase short-acting. *P < 0.05, **P < 0.01, ***P < 0.001.

Figure 5 presents three age bands: under 35 years, between 35 and 38 years, and over 38 years. We conducted a comparative analysis of the differences among the four COS groups within each of these three age categories. In the cycles for women aged <35 years, the highest rates for the four KPIs related to blastocyte development were observed in the LPS GnRH-a group, followed by the FPL GnRH-a group and the GnRH-ant protocol group. The micro-stimulation protocol group showed the lowest rates for all four KPIs.

For patients aged 35–38 and >38 years, the FPL GnRH-a group showed the highest Day 5 BLR/GBLR, total BLR/GBLR as well as implantation rates, followed by the LPS GnRH-a group. Interestingly, the implantation rate was consistently highest in the FPL GnRH-a group, regardless of age, with rates lower in the other two groups. Surprisingly, the Day 5 BLR/GBLR in the micro-stimulation protocol group was higher than that in the GnRH-ant protocol group.

### 3.6 Multivariate regression analysis

MLR analysis showed that implantation rate and KPIs related to blastocyte development, including Day 5 BLR/GBLR, total BLR/GBLR and implantation rate were all significantly associated with female age, number of recovered oocytes, duration of infertility, and COS protocol. The odds ratios (OR), 95% confidence intervals (CI), and p-values for these associations are presented in Table 3. The results were shown by a forest plot (Figure 6) via the R package “ggforestplot”.

**TABLE 3:** Multivariate logistic regression analysis of factors potentially impacting KPIs.

	Day5 good blastulation rate		Day5 blastulation rate		Total good blastulation rate		Total blastulation rate		Implantation rate	
	OR (95%CI)	p value	OR (95%CI)	p value	OR (95%CI)	p value	OR (95%CI)	p value	OR (95%CI)	p value
Female age	0.97 (0.964–0.977)	<0.001	0.976 (0.971–0.983)	<0.001	1.026 (1.019–1.032)	<0.001	0.985 (0.979–0.99)	<0.001	0.954 (0.95–0.959)	<0.001
No. Of recovered oocytes	1.236 (1.228–1.243)	<0.001	1.234 (1.227–1.241)	<0.001	0.807 (0.803–0.812)	<0.001	1.233 (1.226–1.24)	<0.001	0.915 (0.912–0.919)	<0.001
Endometrial thickness	0.998 (0.984–1.013)	0.79	1.005 (0.992–1.018)	0.486	1.003 (0.99–1.017)	0.635	1.002 (0.99–1.014)	0.724	1.037 (1.026–1.049)	<0.001
Female BMI	0.993 (0.983–1.003)	0.174	0.997 (0.988–1.006)	0.482	1.005 (0.995–1.014)	0.341	1.003 (0.995–1.011)	0.457	1.019 (1.012–1.027)	<0.001
Infertility duration	0.982 (0.971–0.992)	0.001	0.977 (0.968–0.986)	<0.001	1.018 (1.008–1.029)	<0.001	0.98 (0.972–0.988)	<0.001	0.985 (0.977–0.993)	<0.001
COS protocol										
MS group	1		1		1		1		1	
FPL group	10.335 (5.801–18.414)	<0.001	8.464 (5.717–12.53)	<0.001	0.131 (0.084–0.204)	<0.001	7.574 (5.719–10.029)	<0.001	127.699 (87.893–185.533)	<0.001
LPS group	2.034 (1.137–3.637)	0.017	1.426 (0.959–2.121)	0.079	0.629 (0.403–0.983)	0.042	1.206 (0.906–1.604)	0.199	69.682 (47.875–101.422)	<0.001
GnRH-ant group	3.422 (1.891–6.192)	<0.001	2.938 (1.959–4.406)	<0.001	0.383 (0.243–0.606)	<0.001	2.971 (2.222–3.973)	<0.001	8.216 (5.607–12.039)	<0.001

Note: OR, odds ratio; CI, confidence interval; FPL, group, follicular phase long-acting GnRH-a, long protocol group; LPS, group, luteal phase short-acting GnRH-a, long protocol group. GnRH-ant, group, GnRH-ant, protocol group; MS, group, micro-stimulation protocol group.

Although endometrial thickness (OR = 1.037; 95% CI: 1.026–1.049, p < 0.001) and female BMI (OR = 1.019; 95% CI: 1.012–1.027, p < 0.001) had a significant effect on the implantation rate, they did not significantly affect KPIs related to blastocyte development.

## 4 Discussion

Infertility is a global issue, affecting approximately 17.5% of adults at some point in their lives (Cox et al., 2022). About 40%–50% of infertile couples seek ART treatments to achieve pregnancy (Boivin et al., 2007). The ART process, which spans from gamete collection to embryo transfer, is complex and influenced by numerous factors. This complexity highlights the importance and challenges of evaluating the quality and efficiency of embryology laboratories. KPIs are essential for implementing new technologies and procedures within the embryology laboratory and for monitoring the efficacy of the quality management system. Structural KPIs primarily assess the foundational elements of the embryology laboratory, including laboratory design, equipment, and staff allocation; process KPIs monitor and control operational procedures, while outcome KPIs evaluate the effectiveness of the IVF laboratory (Fabozzi et al., 2020). Previous studies have established reliable KPIs and explored novel, meaningful KPIs (Hughes and Association of Clinical Embryologists, 2012; Embryology and Medicine, 2017; Vaiarelli et al., 2023; Hammond and Morbeck, 2019; Chamayou et al., 2021). Adjusting KPIs based on age may better monitor embryology laboratory performance (Zacà et al., 2022). In our practice, we observed that the COS protocol influences KPIs; however, there is a lack of research in this area.

The most significant finding in this study was that KPIs differed significantly across the four COS protocol groups, suggesting the potential for adjusting the reference values of these KPIs according to treatment strategies. Notably, the blastocyst development rates and implantation rates in the FPL GnRH-a and the LPS GnRH-a groups were significantly higher than those in the other two groups. This conclusion holds true across nearly all age points and all three age bands (Figures 4, 5). Another key finding is that the BLR and GBLR in the FPL GnRH-a group were higher than those in the LPS GnRH-a group for females under 35 years old; however, the implantation rate in the FPL GnRH-a group was lower than that of the LPS GnRH-a group, regardless of female age.

The BLR/GBLR and implantation rates in the FPL GnRH-a group and LPS GnRH-a group were both higher than those in the other two groups, likely benefiting from the use of GnRH-a. GnRH-a, an analog of hypothalamic Gn-releasing hormone, induces pituitary desensitization and stimulates the development of multiple follicles by promoting synchronized follicular growth. It has been used in IVF procedures for over 40 years (Porter et al., 1984). Both long-acting and short-acting GnRH-a can effectively reduce LH levels on the day of hCG administration, preventing premature luteinization and improving the follicular microenvironment to support follicular development (Siristatidis et al., 2025). GnRH-a not only reduces the fluctuations of endogenous LH and FSH by inhibiting the function of the hypothalamic-pituitary axis, but also produces a flare-up effect, thereby promoting the synchronous development of follicles and improving the implantation rates and pregnancy rates (Wang et al., 2023; Luo et al., 2020). Overall, there is limited articles comparing GnRH-a regimens with micro-stimulation protocols. Youssef (Youssef et al., 2017) et al. found that a mild ovarian stimulation strategy for women with poor ovarian reserve undergoing IVF results in ongoing pregnancy rates comparable to those achieved with conventional ovarian stimulation strategies. However, several studies align with our findings, demonstrating that GnRH-a protocols yield higher implantation rates and blastocyst development rates compared to other protocols (Lv et al., 2022; Huang et al., 2018).

In the two groups that did not use GnRH-a, our study revealed significantly higher BLR/GBLR and implantation rates in the GnRH-ant protocol group compared to the micro-stimulation group for individuals under 35 years of age. GnRH-antagonists inhibit the early onset of the LH peak during the middle and late stages of follicular growth, which prevents the suppression of endogenous LH and FSH during the early follicular phase, thereby promoting follicular development during the initial recruitment phase and preserving pituitary responsiveness (Zhang et al., 2021). However, micro-stimulation protocols involve shorter stimulation cycles and necessitate lower doses of gonadotropins. Although no statistically significant difference was observed in subgroups over the age of 35, a consistent trend was noted. This observation warrants further investigation in future studies.

In addition, we observed that the Day 5 BLR/GBLR and total BLR/GBLR in the LPS group prior to the age of 35 were both higher than those in the FPL group (Figures 4, 5). However, there is little literature directly addressing this concern. We hypothesize that this may be related to the lower LH levels on the day of hCG administration in the LPS GnRH-a group. Interestingly, regarding the blastocyst development rates, we observed almost the opposite conclusion in cycles after the age of 35, which currently lacks strong supporting evidence and awaits further exploration in other studies. LH, a crucial hormone secreted by the anterior pituitary, stimulates oocyte maturation and plays a key role in ovulation and corpus luteum maintenance (Ezcurra and Humaidan, 2014; Orisaka et al., 2013). LH and FSH work synergistically to support normal follicular development (Orvieto et al., 2021), with low LH levels negatively affecting oocyte retrieval and fertilization rates (Dragotto et al., 2024; Makolle et al., 2021).

Unexpectedly, a previous review found no significant difference between depot and daily GnRH-a use for pituitary down-regulation in IVF cycles with the long protocol (Albuquerque et al., 2013), suggesting that variations in blastocyst formation rates may not be directly related to LH levels. However, more research supports our ideas. Numerous studies have shown that timely administration of LH can enhance pregnancy outcomes (Alviggi et al., 2018; Benmachiche et al., 2019; De Placido et al., 2005; Bosch et al., 2011). The half-life of short-acting GnRH-a is shorter than that of long-acting GnRH-a, resulting in more rapid recovery of pituitary desensitization and higher LH levels, within the LH window (Balasch et al., 2001). Excessive suppression of the pituitary can lower LH levels, interfering with paracrine signaling between granulosa and theca cells, which affects estrogen and androgen synthesis, preventing complete oocyte maturation (Verpoest et al., 2000).

Our study provides evidence supporting that the implantation rate was significantly higher in the GnRH-a treatment groups compared to the other two protocols. Besides, we observed that between the two groups using GnRH-a, the FPL GnRH-a group had a higher implantation rate. GnRH-a treatment can improve oocyte quality, regulate cytokine secretion, and enhance endometrial receptivity and the pelvic microenvironment (Cao et al., 2020; Kolanska et al., 2017; Xu et al., 2020), facilitating embryo implantation. Previous studies have suggested that GnRH-a may decrease estrogen production and enhance endometrial repair capacity by upregulating Tff1 and Sprr2a1 (Guo et al., 2017). A possible explanation for the higher implantation rate in the FPL GnRH-a group may be the decreased resistance in sub-endometrial blood flow, along with the resulting increase in endometrial thickness. Consistently, previous studies have shown that GnRH-a prolonged protocol is superior to short GnRH-a long protocol in terms of clinical pregnancy rates and implantation rates, particularly in patients with thinner endometria (Song et al., 2020). Xu (Xu et al., 2020) et al. suggested that depot GnRH-a may protect the expression of HOXA10, MEIS1, and LIF, positively impacting endometrial receptivity and leading to higher live birth rates.

Based on the findings of this study, we conclude that the LPS GnRH-a long protocol results in the highest total GBLR, while the FPL GnRH-a long protocol provides the highest implantation rates. COS protocols can be tailored according to the patient’s needs. For patients who do not require fresh embryo transfer, such as those needing PGD/PGS or embryo freezing, the LPS GnRH-a long protocol may be used to increase blastocyst yield. For patients requiring fresh embryo transfer, the FPL GnRH-a long protocol should be used to improve implantation rates. For patients over 40 years old, the results of the four COS protocols are similar, and the GnRH-ant or micro-stimulation protocols, which are more cost-effective and shorter in duration, can be considered.

Additionally, our results support the role of female age in embryo and blastocyst quality and implantation rates. Extensive research has shown that advancing maternal age adversely affects embryo development and transfer outcomes. Studies indicate that older maternal age is associated with an increased incidence of aneuploidy in oocytes and embryos, increased mitochondrial DNA damage (Coughlan et al., 2014), and higher rates of embryo-endometrial asynchrony (Shapiro et al., 2016), all of which contribute to reduced implantation rates.

Utilizing KPIs for embryo laboratory quality control is crucial for total quality management (De los Santos et al., 2016), and previous guidelines and consensus have provided robust KPIs with agreed competency levels and benchmark values (Hughes and Association of Clinical Embryologists, 2012; Embryology and Medicine, 2017; Vaiarelli et al., 2023). KPIs play a vital role in introducing laboratory technologies or operational processes, establishing minimum performance standards, monitoring the effectiveness of the quality management system, and assessing quality improvements. The efficacy of KPIs depends on the accurate and comprehensive collection of data, and their reasonable utilization is essential to enhance safety, efficacy, and efficiency (Malhotra et al., 2013). The blastocyst/good embryo development rates and implantation rate studied here are common and effective KPIs used in embryo laboratories. Given the observed differences in these KPIs across the four COS protocols, we believe it is necessary to adjust the reference values for these KPIs according to treatment protocols. Although we observed that KPIs varied among COS groups, contributing to a more rational evaluation of embryo laboratory efficacy, we do not assert that these protocols are inherently superior or inferior. The choice of treatment regimen should be informed by a variety of factors. We recommend considering total BLR, total GBLR, and implantation rate as potential KPIs to evaluate the laboratory efficacy of different COS protocols. However, the incorporation of age into the assessment and the determination of appropriate reference values warrant further investigation.

This study’s major limitation is its retrospective nature, which introduces potential biases and does not eliminate individual differences. Additionally, there was considerable variation in sample sizes among the four COS groups. As this is a single-center study, future multicenter studies with larger sample sizes are needed to validate our findings. While our study demonstrated differences in KPIs across treatment groups, it does not provide explicit reference values for each COS protocol. However, this approach contributes to refining our understanding of how KPIs can be adjusted to improve the quality control of embryology laboratories. This may provide a theoretical basis for personalized COS protocols for patients, and offer novel suggestions for forecasting ART laboratory outcomes.

## 5 Conclusion

In summary, COS protocols significantly influence KPIs, indicating that adjusting the reference values of these indicators according to the treatment protocols may help optimize the quality control of ART laboratories. Our study also supports the effectiveness of the KPIs recommended in previous consensus guidelines. COS protocols can be tailored based on the patient’s individual needs. Further well-designed, multicenter studies are necessary to validate these results and establish explicit reference values for each COS protocol.

## Data Availability

The original contributions presented in the study are included in the article/supplementary material, further inquiries can be directed to the corresponding author.

## Abbreviations

BLR, blastocyst development rate; D5BUR, Day 5 Usable Blastocyst Rate; FPL, follicular phase long-acting; GBLR, good blastocyst development rate; LPS, luteal phase short-acting.
